# Intersecting identities, different struggles: The effects of demographics on experiences of discrimination and mental health outcomes among college students in Texas

**DOI:** 10.1371/journal.pmen.0000425

**Published:** 2025-09-04

**Authors:** Amanda Venta, Jesse Walker, Daniel O’Connell, Tessa Long, Cynthia M. Navarro Flores, Alejandro L. Vázquez

**Affiliations:** 1 Department of Psychology, University of Houston, Houston, Texas, United States of America; 2 Department of Psychology & Philosophy, Sam Houston State University, Huntsville, Texas, United States of America; 3 Department of Psychology & Neuroscience, The University of Tennessee, Knoxville, Tennessee, United States of America; UCL: University College London, UNITED KINGDOM OF GREAT BRITAIN AND NORTHERN IRELAND

## Abstract

Diversity among college students has increased and, yet experiences of discrimination persist and predict mental health symptoms. The role of various overlapping demographics in predicting discrimination and mental health is understudied. In this study, we examined how gender,race, Latinx ethnicity, and citizenship status relate to experiences of discrimination and mental health outcomes in *N* = 1665 college students in Texas. Our results indicated that discrimination was endorsed at higher rates by People of Color, Latinxs, women, and non-citizens. Women and non-citizens endorsed the highest mental health symptoms. Multivariate analyses demonstrated greatest vulnerability for discrimination among Latinx women. Regarding mental health, experiences of discrimination associated with stigmatization were associated with deleterious mental health outcomes, particularly for Women of Color. Reflecting the growing scholarship on intersectionality, race and Latinx ethnicity acted with gender to compound risk for discrimination and the effect of discrimination on mental health.

## Introduction

Discrimination and college student mental health is more relevant than ever given the increasing diversity of college students in the United States (U.S.). People of Color now make up 44.8% of U.S. college students, and female enrollment has grown, including enrollment of Latinx (The term Latinx is utilized throughout this manuscript in an effort to be as inclusive as possible regarding gender diversity and in accordance with the guidance of the National Latinx Psychological Association (Cardemil, Millán, & Aranda, 2019).) and Black women [1]. Growing diversity is further demonstrated in the increase of immigrant students—who make up 28% of all college students—and non-citizen students (with an estimated 2% or 454,000 being undocumented; [2]. In Texas, Latinx students are the most represented racial/ethnic group (40.6%) and significantly more women than men are enrolled [3]. Still, Texas remains politically conservative at the state level and recent state laws—like Texas Senate Bill 17 that targets Diversity, Equity, and Inclusion activities at public institutions of higher education, which stands to increase feelings of marginalization among students of color as well as contribute to xenophobic, anti-immigrant, and racist attitudes and actions in the form of harassment and discrimination [4]. The aim of the present study was to examine experiences of discrimination and mental health among college students in Texas, with a particular focus on how race, ethnicity, gender, and citizenship predict experiences of discrimination and the effects of discrimination on mental health.

College students who belong to marginalized social groups (e.g., racial [group defined by shared physical characteristics]/ ethnic minorities [group defined by shared culture, values, beliefs, practices], LGBTQ + , immigrants, women) often experience, minority stress, or chronic and unique stressors arising from systemic discrimination, prejudice, and social exclusion [5]. Minority stress is composed of proximal (e.g., prejudice) and distal processes (e.g., expectations of rejection, concealment, internalize homophobia) that impact health outcomes [5]. The accumulation of these stressors across time can negatively impact mental and physical health minoritized college students [5].

Understanding impacts of minority stress on health outcomes of minoritized college students requires consideration of intersectional identities that can create heightened or different stress experiences based on interactions with interlocking systems of oppression (e.g., racism, xenophobia, heterosexism). Our approach is guided by scholarship on “intersectionality,” a concept introduced nearly thirty years ago to describe how race and gender interact to shape identity and experiences of discrimination [6,7]. Indeed, Crenshaw [6] noted that “Black women sometimes experience discrimination in ways similar to White women’s experiences; sometimes they share very similar experiences with Black men. Yet often they experience double-discrimination, discrimination as Black women—not the sum of race and sex discrimination, but as Black women”. While the intersectionality framework emphasizes “systems and overlapping structures of power, privilege and oppression” [8]—rather than simply intersecting identities or demographics—Crenshaw [6] underscored how identities such as being both Black and female are critical to understanding discrimination. From a systemic perspective, these overlapping identities create simultaneous experiences of oppression [9]. Rooted in Black feminism and Critical Race Theory, intersectionality theory serves as an analytical tool to examine how oppression and marginalization operate in society through individuals’ intersecting identities [10]. While initially applied to the experiences of Black women, scholars have extended this concept to include other overlapping identities (e.g., gender, ethnicity, sexual orientation) to understand the experiences of other marginalized groups and the power structures at play (e.g., sexism, racism; [10]. By “marginalized”, we refer to individuals who are disadvantaged and oppressed in contrast to more privileged individuals, due to the social construction of diversity and difference. Marginalized identities are excluded rather than included, devalued rather than valued, and oppressed rather than elevated compared to privileged identities [11].

In this study, we use the ADDRESSING Model to guide the identities that are considered marginalized versus privileged [12]. The ADDRESSING Model considers various intersecting identities (i.e., Age/generation, Disability status, Religion/spirituality, Ethnicity/race, Socioeconomic status, Sexual orientation, Indigenous heritage, Nationality, Gender) that interact with systems of oppression shape health outcomes among people of color [12]. Specifically, we focus on race and ethnicity, where People of Color (e.g., Latinx) are marginalized; gender, where women are marginalized compared to men; and citizenship, where U.S.-born individuals are privileged relative to non-citizens. Specifically, we sought to understand how the intersection of these identities contributes to disadvantage, particularly in the form of individuals’ experiences of discrimination and their impact on mental health among college students in Texas.

### Experiences of discrimination

Though U.S. colleges continue to diversify, discrimination–defined as being treated unfavorably due to belonging to a specific group [13,14]—continues, with growing evidence of its negative impacts [15–17]. Discrimination negatively affects learning and overall quality of the educational experience [18]. Socially, discrimination contributes to feelings of invisibility, isolation, and disconnection [19–23]. These experiences are more pronounced at primarily White institutions [24]. Experiences of discrimination differ across identities. Specifically, Latinxs endure discrimination regarding non-citizen immigration status or having an accent [25,26] and may struggle to or decide not to advocate for themselves due to documentation status, language barriers, and/or limited knowledge of social structures [27]. These experiences have worsened in recent years with increases in anti-immigrant rhetoric and policies within the U.S. including vilification of immigrants, increased deportations and family separations, and attempts to end programs that provide legal immigration status for youths brought to the U.S. as children such as Deferred Action for Childhood Arrivals (DACA) program [28]. For Black Americans, experiences of discrimination are more prevalent and likely to be race-related, with some reporting experiencing discrimination up to once a week [24,29].

### Discrimination and mental health

Experiences of discrimination can take a toll on mental health [25,30], particularly anxiety and depression [24,31,32], and effects vary demographically. For instance, Latinx ethnicity is associated with higher rates of discrimination and mental health symptoms compared to White and Asian American students [24]. However, this vulnerability is further moderated by gender, with Latinx women being more likely to experience depression that is associated with discrimination compared to Latinx men [33]—though research is mixed [34]. Likewise, relations between discrimination and psychological distress are stronger among Black women than men [35]. Non-citizen immigration status, particularly undocumented status, has been repeatedly linked to increased mental health problems in adults from Latinx and other ethnic backgrounds [36]. Moreover, gender moderates the relation between discrimination and anxiety in college students of color [37]. Notably, the mental health impacts of discrimination stretch to critical public health crises, with discrimination being correlated to suicide-related thoughts and behaviors among college students of color, Latinxs, and non-citizens [20,24,33,38–40]. These mental health problems stemming from discrimination experiences can also impact the academic performance of minoritized college students by affecting their grades, likelihood of graduating on time, and less school satisfaction [41]. As a result, minoritized college students facing discrimination may struggle to achieve their full academic potential, which could limit their long-term economic mobility and health [41,42].

### The present study

The existing literature possesses notable gaps. Specifically, research on the impacts of discrimination in Latinxs is limited [43,44] and there is a paucity of research exploring the impacts of citizenship status on mental health in the context of discrimination [43,44] with limited data including Latinxs [25,26]. Relevant to the current study, most research does not consider overlapping demographics in relation to discrimination and mental health outcomes, meaning that the extant literature does not adequately represent U.S. college campuses that are now occupied by individuals that cut across demographic lines (e.g., Black, Latinx, non-citizen, woman). While racial diversity within Latinxs has garnered some attention in recent scholarship [45], citizenship status is rarely considered outside of Latinxs and, to our knowledge, has not been considered alongside race and gender, failing to recognize the overlap of these demographic characteristics. Moreover, Latinxs in Texas—a hotbed of anti-immigrant rhetoric and policy—are understudied.

The aim of the present study was to examine how demographics among college students in Texas relate to discrimination and mental health. Our approach was guided by cultural and minority stress theories [12,46], alongside the intersectionality framework focusing on understanding how multiple intersecting identities interact and result in varying experiences of oppression and marginalization [9]. These theories informed our global hypothesis that participants who identify with demographic groups that have been oppressed in the U.S. would report increased experiences of discrimination and, in turn, evidence a significant relation between these experiences and mental health symptoms. Our analytic approach centered on modeling interactions between demographic identities (e.g., Latinx and gender) in order to capture the lived experiences of these unique groups via statistical variance in outcomes of interest—discrimination and mental health—accounted for not only by being, for example, a Person of Color or a woman, but also by being a woman of color, in line with recent scholarship on disaggregating demographic identities [47].

In this study, we focused on the social context of Texas—a diverse state with a high proportion of Latinx (39.3%) and Black (11.8%; [48]) Americans—and the state with the second highest concentration of undocumented immigrants (5.7%; [49]). Our first aim was to assess the contributions of race, Latinx ethnicity, gender, and citizenship status on experiences of discrimination among college students. We hypothesized that People of Color, Latinxs, women, and non-citizens would report increased discrimination (Hypothesis 1A; H1A). Moreover, we expected to find significant two-way interactions indicating that Women of Color, Latinx women, Latinx People of Color, female non-citizens, and non-citizens of color would experience greater discrimination (Hypothesis 1B; H1B). (We did not expect to find significant interaction effects of Latinx ethnicity and citizenship status given the overlap of these groups in Texas.) We also expected to find significant three-way interactions indicating that People of Color, Latinx women, and female non-citizens of color experience greater discrimination than other groups (Hypothesis 1C; H1C). Our second aim was to assess the effects of discrimination on mental health outcomes, hypothesizing that discrimination would be associated with negative mental health outcomes, and that these effects would be exacerbated by demographic factors. Specifically, we hypothesized significant two-way interactions of discrimination with race, Latinx ethnicity, female gender, and non-citizen status in predicting negative mental health outcomes such that the relation between discrimination and mental health symptoms would be stronger in People of Color, Latinxs, women, and non-citizens (Hypothesis 2A; H2A). More complex interactions were exploratory in nature. Our study aimed to add to the existing literature on discrimination among college students in Texas by computing demographic interaction terms and testing their effects on experiences of discrimination and mental health.

## Materials and methods

### Ethics statement

Data were collected as a part of a larger study examining the mental health of students across seven separate Texas universities between May 30^th^, 2017 – December 31^st^, 2018. All recruitment sites were four-year, public universities but differed in terms of demographics, population density, and location. Institutional Review Board (IRB) approval was obtained from Sam Houston State University (protocol # 2017-04-34345). Participants were recruited through each institution’s online research platform and received extra course credit. Consent was indicated by reading a cover letter and proceeding to the study battery. Participation was completely voluntary and anonymous, and participants were able to decline at any time without penalization. The full study battery was approximately 90 minutes.

### Participants

The complete sample included 3158 participants. Two participants were removed due to being outside of the IRB-approved age range (that is, they participated in the survey despite being under 18 years of age and able to provide consent). Three questions functioning as attention checks required participants to select a specific answer (e.g., “Choose ‘Somewhat agree’ as your answer”). Participants who provided any other answer (i.e., incorrect answers) to the attention check questions were removed, leaving *n* = 1973. The large number of participants who failed the attention checks is to be expected with online data collection. Missing data across study variables (not mutually exclusive) was as follows: **n* *= 9 for gender, **n* *= 4 for race, **n* *= 17 for ethnicity, **n* *= 209 for citizenship status, **n* *= 22–30 for experiences of discrimination, and **n* *= 11–14 for mental health outcomes and was excluded in a pairwise fashion. In addition, because only two participants endorsed a gender other than male or female, they were excluded. Participants were excluded in a pairwise fashion for a final sample of 1665 participants.

Participants were enrolled as undergraduate students and ranged from 18 and 59 years (*M* = 21, *SD* = 4.42), with 82% being between ages 18–22. No participant was excluded based on age to maximize generalizability to the typical college sample, in which the mean age is 26.4 years (U.S. Department of Education, 2012). Participant characteristics appear in Table 1. To minimize small cell sizes, participant race was recoded into People of Color (**n* *= 927, 48.2%) or White (**n* *= 995, 51.8%) and immigration status into citizen (**n* *= 1418, 80.4%) and non-citizen (**n* *= 346, 19.6%).

**Table 1:** Participant characteristics.

Identity Variable	Participants Endorsed	
	*Percentage*	*n*
Female	75.2%	1478
Latinx	55.4%	1084
Race: White	51.8%	927
Race: Black	12.5%	240
Race: Asian	6.6%	127
Race: Native Hawaiian or Pacific Islander	0.5%	9
Race: American Indian or Alaskan Native	2.3%	44
Race: Mixed or other	26.4%	507
Immigration: Citizen	80.4%	1418
Immigration: Legal permanent resident	6.6%	117
Immigration: Deferred Action for Childhood Arrivals	7.1%	126
Immigration: visa/green card holder	4.7%	83
Immigration: refugee/asylee	0.1%	1
Immigration: Undocumented	1.1%	19

### Measures

#### Demographic form.

Investigators wrote a specific questionnaire capturing relevant demographic information. This form included questions on age, gender, race, Latinx ethnicity, and citizenship status.

#### Discrimination.

The Brief Perceived Ethnic Discrimination Questionnaire (PEDQ; [50] is a 17-item self-report survey which assesses experiences of discrimination regarding exclusion, workplace discrimination, stigmatization, and threat and harassment. All participants were asked to complete the PEDQ, even if they identified as belonging to majority ethnic and racial groups, given prior research suggesting the presence of perceived anti-White bias among young adults [51,52]. For our purposes, the instructions were modified to ask respondents to answer questions based on their “group” (i.e., “how often have any of the things listed below ever happened to you, because of the group that you belong to?”) without specifying whether “group” referenced gender, ethnic, racial, or other identity characteristics. Sample items on this instrument include: “have others thought you couldn’t do things or handle a job?” (Workplace); “have others threatened to hurt you (ex: said they would hit you)?” (Threat); “have others hinted that you are dishonest or can’t be trusted?” (Stigma), and “have others made you feel like an outsider who doesn’t fit in because of your dress, speech, or other characteristics related to your ethnicity?” (Exclusion). Responses range from [1] *never* to [5] *very often***.** PEDQ items were averaged with higher scores representing great symptom severity. Limitations of this measure include that the type and setting of discrimination are not assessed and that some questions query ethnic discrimination specifically. Cronbach’s alphas for the PEDQ subscales in this study were: Exclusion.68, Workplace.83, Stigma.79, and Threat.83, which aligns with previous research [50]. Importantly, the measure has been used reliably in Latinx and Black samples [33,50]. Evidence of the PEDQ’s validity is drawn from significant between-group differences, with Black and Latinx respondents indicating greater discrimination than White respondents, as well as within-group variation among Latinxs, with immigrant Latinxs reporting more discrimination [50]. Validity analyses link the PEDQ with measures of perceived racism as well as experiences of discrimination reported by participants over the previous week [50].

#### Mental health.

The Depression Anxiety and Stress Scale (DASS; [53] is a 21-item self-report questionnaire which assesses a range of symptoms experienced over the last week. It features three subscales, depression, anxiety, and stress, and exhibits high levels of reliability (Lovibond & Lovibond, 1995). Sample items on this scale include: “I experienced breathing difficulty (e.g., excessively rapid breathing, breathlessness in the absence of physical exertion),” “I felt down-hearted and blue,” and “When I’m upset, it takes me a long time to feel better.” Response ranged from [1] *almost never* to [5] *almost always*. DASS items were summed with higher scores representing great symptom severity. Cronbach’s alphas for the DASS subscales in this study were as follows: Depression.91, Anxiety.82, and Stress.85. The questionnaire has demonstrated adequate validity in diverse samples, including Black and Latinx populations [54,55], with evidence of internal consistency, convergent, and divergent validity across racial and ethnic groups.

### Data analytic strategy

We report how we determined our sample size, all data exclusions, all manipulations, and all measures in this study. Data are available via email from the corresponding author. This study was not preregistered. Bivariate analyses were conducted to characterize relations between main study variables and identity covariates (H1A; H2A). The assumptions of MANCOVA were examined in preliminary analyses. First, outliers in PEDQ and DASS subscales were examined utilizing box plots and excluded. This resulted in excluding data for four participants on the PEDQ and none on the DASS. Second, normality was evaluated utilizing the Shapiro-Wilk statistic which indicated non-normality in the PEDQ and DASS subscales (all *p* < .001). Given unreliability in this statistic in large samples [56], normality was also evaluated utilizing skewness and kurtosis, which was acceptable (skewness ≤ 2, kurtosis ≤ 7; [56] for all outcomes except the Threat subscale (skewness = 3.75, kurtosis = 17.13). Logarithmic and square root transformations were unsuccessful in addressing non-normality in the Threat subscale. Third, linearity and non-multicollinearity were assessed via the correlations reported in Table 1, which indicated significant, linear relationships among dependent variables that did not exceed **r* *= .90 [57]. Finally, homogeneity of variance was assessed utilizing Box’s M statistic, which was significant with PEDQ subscales and with DASS subscales as outcome variables (*p* = .004) indicating violation of this assumption. Thus, Pillai’s Trace values, which are also the most robust to non-normality as in the Threat variable [58], were reported.

For each aim, one MANCOVA model was run to control familywise Type 1 error, and follow-up, post-hoc analyses were Bonferroni corrected [59]. Specifically, in service of aim 1, a MANCOVA was examined in which the four PEDQ subscales were entered as outcome variables; gender, race, Latinx ethnicity, citizenship status, and all two-, three-, and four-way interactions were entered as independent variables; financial aid status and university recruitment site served as covariates (H1A-C). In service of the second aim, a second MANCOVA was examined in which the three DASS subscales were entered as outcome variables; gender, race, Latinx ethnicity, citizenship status, and the four PEDQ variables as well as two-, three-, and four-way interactions were entered as independent variables; and age served as a covariate (H2A). Post-hoc pairwise comparisons and simple-slope comparisons were conducted if relevant. A priori power analyses were not conducted for this study. Observed power was computed for each main and interactive effect and only effects with observed power equal to or greater than.8 were reported. Analyses were conducted in SPSS version 23.

## Results

### Bivariate analyses

Races differed across all PEDQ variables (H1A), such that higher discrimination was endorsed by participants of color: Exclusion, *t* (1790.45), = 5.86, **p* *< .001, **d* *= 0.27, Workplace, *t* (1806.37), = 8.67, **p* *< .001, **d* *= .40, S*t*igma, *t* (1769.15), = 7.14, **p* *< .001, **d* *= .33, and Threat, *t* (1693.85), = 3.62, **p* *< .001, **d* *= 0.17. Men and women differed significantly regarding Exclusion, *t* (1940), = -2.51, **p* *= .012, **d* *= .13, and Workplace, *t* (890.05), = -3.37, **p* *< .001, **d* *= .17, with women endorsing higher discrimination. Par*t*icipan*t*s also differed on experiences of discrimination depending on Latinx ethnicity: Exclusion, *t* (1932) = 2.72, **p* *= .007, **d* *= .12, Workplace, *t* (1907.21) = 6.06, **p* *< .001, **d* *= .28, and Stigma, *t* (1899.38) = 3.10, **p* *= .002, **d* *= .14. Likewise, all four subscales of the PEDQ differed according *t*o ci*t*izenship, with greater discrimina*t*ion reported by non-citizens: Exclusion, *t* (478.76), = 3.11, **p* *= .002, **d* *= .19, Workplace, *t* (472.79), = 4.64, **p* *< .001, **d* *= .29, Stigma, *t* (461.65), = 3.12, **p* *= .002, **d* *= .20, and Threat, *t* (455.94), = 2.43, **p* *= .016, **d* *= .16.

Regarding mental health, race groups differed by Stress: *t* (1956), = -2.45, **p* *= .014, **d* *= 0.11, such that White respondents reported more stress. Men and women differed significantly regarding mental health: Depression, *t* (1951), = -3.43, **p* *= .001, **d* *= 0.18, Anxiety, *t* (928.57), = -7.29, **p* *< .001, **d* *= 0.37, and Stress, *t* (914.55), = -7.14, **p* *< .001, **d* *= 0.36, with higher symptoms among women. Latinx ethnicity groups differed by stress, *t* (1943), = -2.26, **p* *= .024, **d* *= 0.10. Citizenship s*t*atus groups differed across mental health symptoms across variables: Depression, *t* (1752), = 2.20, **p* *= .028, **d* *= 0.13, Anxiety, *t* (1749), = 2.72, **p* *= .007, **d* *= 0.16, and Stress, *t* (1752), = 1.93, **p* *= .053, **d* *= 0.11, with higher symp*t*oms among non-citizens.

Correlations between continuous variables are presented in Table 2 (H2A). Moderate, significant correlations were evident between DASS and PEDQ. Exploratory bivariate analyses were undertaken to determine whether university recruitment site, financial aid receipt, and age should be included as covariates. Participants from the varied university recruitment sites (nominal variable) differed according to Exclusion, *F* (6, 1921) = 4.50, **p* *< .001, Workplace, *F* (6, 1915) = 5.64, **p* *< .001, and Stigma, *F* (6, 1924) = 3.62, **p* *= .001. Participants receiving financial aid (nominal variable) differed from those who did not with regard to Workplace, *t* (1088.57), = 2.56, **p* *= .011, **d* *= .13, and Stigma, *t* (1125.16), = 2.19, **p* *= .028, **d* *= .11. Therefore, financial aid receipt and universi*t*y recruitment site were included as nominal covariates when examining PEDQ. Age did not demonstrate significant relations to discrimination (**p* *= .172-.886).

**Table 2:** Correlations between continuous study variables.

Outcomes	Age	PEDQ Exclusion	PEDQ Workplace	PEDQ Stigma	PEDQ Threat	DASS Depression	DASS Anxiety
PEDQ Exclusion	-0.003	1.00					
PEDQ Workplace	-0.01	.76***	1.00				
PEDQ Stigma	-0.02	.75***	.75***	1.00			
PEDQ Threat	-0.02	.58***	.51***	.54***	1.00		
DASS Depression	-.11***	.24***	.27***	.25***	.22***	1.00	
DASS Anxiety	-.12***	.24***	.26***	.23***	.22***	.68***	1.00
DASS Stress	-.08***	.27***	.29***	.26***	.24***	.82***	.76***

*Notes.* PEDQ = Perceived Ethnic Discrimination Questionnaire; DASS = Depression Anxiety and Stress Scale. * p < 0.05, ** p < 0.01 and *** p < 0.001.

Regarding mental health outcomes, financial aid status groups did not differ with regard to Depression, *t* (1945) = -0.81, **p* *= .421, Anxiety, *t* (1942), = -0.34, **p* *= .733, or Stress, *t* (1945) = 0.06, **p* *= .949. University recruitment site groups did not differ with regard to Depression, *F* (6, 1932) = 1.31, **p* *= .249, Anxiety, *F* (6, 1929) = 0.65, **p* *= .689, or Stress, *F* (6, 1932) = 1.96, **p* *= .068. Therefore, financial aid and university recruitment site were not included as covariates in analyses related to mental health, but age was.

### Aim 1: Effects of gender, race, Latinx ethnicity, citizenship status, and their overlap on experiences of discrimination

A MANCOVA was conducted utilizing all PEDQ variables as outcomes; gender, race, Latinx ethnicity, citizenship status, and their interactions as predictors; and financial aid status and university recruitment site as covariates. At the multivariate level two significant main effects and one interactive effect emerged: gender main effect, *F* (4, 1647) = 4.26, *p *= .002, *η*^*2*^ = .01, *power* = .93; Latinx ethnicity main effect, *F* (4, 1647) = 2.40, *p *= .048, *η*^*2*^ = .01, *power* = .80; and gender*Latinx ethnicity interaction, *F* (4, 1647) = 2.98, *p *= .018, *η*^*2*^ = .01, *power* = .80. Multivariate effects were probed via tests of between subjects effects. Specifically, significant between-subjects effects of gender*Latinx ethnicity were noted regarding the Exclusion, *F* = (1) 5.68, *p *= .017, *η*^*2*^ = .003, Workplace, *F* (1) = 7.92, *p *= .005, *η*^*2*^ = .01, and Stigma *F* (1) = 11.40, *p *= .001, *η*^*2*^ = .007, subscales. Post-hoc Bonferroni corrected comparisons indicated that Exclusion related discrimination was rated highest by Latinx women (*M* = 6.42, *SD* = 2.56)—significantly higher than non-Latinx women (*M* = 5.98, SD = 2.42; **p* *= .006) and non-Latinx men (*M* = 5.87, *SD *= 2.40; *p* = .012)—but not significantly different from Latinx men (*M* = 5.96, *SD* = 2.41; *p* = .087). Likewise, post-hoc Bonferroni corrected comparisons indicated that Workplace related discrimination was rated highest by Latinx women (*M* = 7.34, *SD *= 2.84)—significantly higher than non-Latinx women (*M* = 6.25, *SD *= 2.89; *p* < .001), non-Latinx men (*M* = 6.40, *SD *= 2.92; *p* < .001), and Latinx men (*M* = 6.34, *SD *= 2.84; *p* < .001). However, post-hoc Bonferroni corrected comparisons revealed a different pattern for Stigma related discrimination, which was rated highest by Latinx men (*M* = 5.89, *SD *= 2.63)—significantly higher than non-Latinx women (*M* = 5.33, *SD *= 2.16; *p* = .014) but not significantly different from non-Latinx men (*M* = 5.61, *SD *= 2.42; *p* = 1.000) or Latinx women (*M* = 5.88, *SD *= 2.51; *p* = 1.000). Additionally, Latinx women rated this subscale significantly higher than non-Latinx women (*p* = .014).

### Aim 2: Relation between experiences of discrimination and mental health

A MANCOVA was conducted utilizing all three DASS variables as outcome variables; PEDQ variables, gender, race, Latinx ethnicity, citizenship status, and their interactions as predictor variables; and age as a covariate. At the multivariate level, there was a significant interaction of gender, race, and Stigma, *F* (3, 1563) = 4.75, *p *= .003, *η*^*2*^ = .01, *power *= .90, on mental health. Simple slopes for the association between Stigma related discrimination and mental health were tested across gender*race groups. Regarding Depression, each of the simple slope tests revealed a significant, positive association between discrimination and depression, with the strength of the effect as follows, from highest to lowest: Women of Color (*B* = .54, *SE* = .06, *p* < .001), White men (*B* = .47, *SE *= .13, *p* = .004), White women (**B* *= .40, *SE* = .08, *p* < .001), and Men of Color (*B* = .28, *SE *= .11, *p* = .009). Fisher z transformations were used to conduct pairwise comparisons of these slopes utilizing a corrected alpha value equal to.008 (.05/6). After applying this correction, the following statistically significant differences were observed: the relation between discrimination and depression was significantly greater in women of color than in white women (*z* = 3.42, **p* *< .001) and men of color (*z* = 4.16, *p* < .001), and the relation between discrimination and depression was significantly greater in white men than in men of color (*z* = 2.4, *p* = .008).

Regarding Anxiety, each of the simple slope tests revealed a significant, positive association between discrimination and anxiety, with the strength of the effect as follows, from highest to lowest: Women of Color (*B* = .39, *SE *= .05, *p* < .001), White women (*B* = .38, *SE *= .07, *p* < .001), Men of Color (*B* = .32, *SE *= .09, *p* < .001), and White men (*B* = .31, *SE *= .10, *p* = .003). Fisher z transformations were used to conduct pairwise comparisons of these slopes utilizing a corrected alpha value equal to.008 (.05/6). No pairwise comparisons were significant following correction.

**Fig 1:**
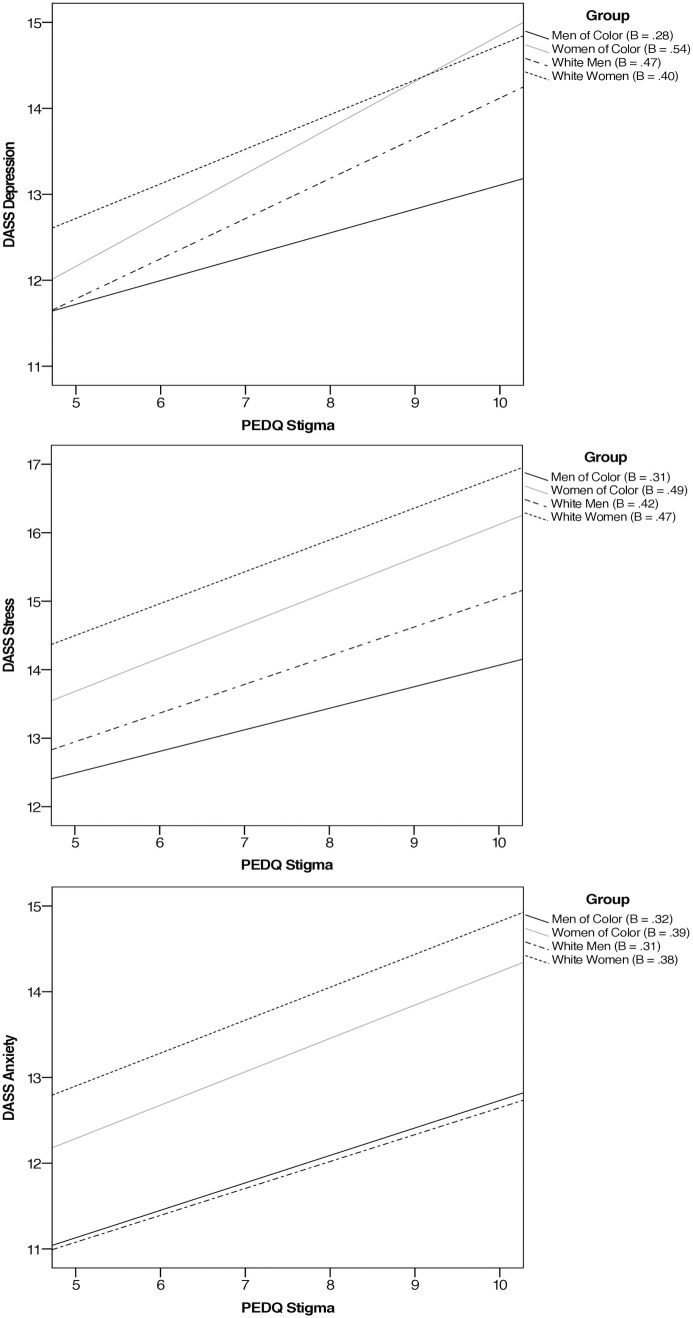
Linear relations between PEDQ stigma and mental health outcomes.

Regarding Stress, each of the simple slope tests revealed a significant, positive association between discrimination and stress, with the strength of the effect as follows, from highest to lowest: Women of Color (*B* = .49, *SE *= .06, *p* < .001), White women (*B* = .47, *SE *= .07, *p* < .001), White men (*B* = .42, *SE *= .11, *p* < .001), and Men of Color (*B* = .31, *SE *= .09, *p* < .001). Fig 1 plots the simple slopes for these interactions. Fisher z transformations were used to conduct pairwise comparisons of these slopes utilizing a corrected alpha value equal to.008 (.05/6). After applying this correction, the following statistically significant differences were observed: the relation between discrimination and stress was significantly greater in women of color than in men of color (*z* = 2.38, **p* *= .002), and the relation between discrimination and stress was significantly greater in white women than in men of color (*z* = 2.5, *p* = .006).

## Discussion

The broad aim of the current study was to examine how the demographic characteristics of college students in Texas relate to experiences of discrimination and mental health. Our findings replicated prior research documenting increased discrimination among women [33], People of Color [24], Latinxs [24], and non-citizens [36]. Overall, our findings pointed to the importance of considering both gender and race/ethnicity in understanding experiences of discrimination and their effects on mental health. Indeed, multivariate analyses indicated particular vulnerability for discrimination among Latinx women. Regarding mental health outcomes, our findings indicate increased mental health symptoms among women [33] and non-citizens [36] in college as well as relations between mental health symptoms and discrimination. Multivariate analyses indicated particular vulnerability for mental health problems among Women of Color experiencing discrimination.

Our analytic approach explains why many of the overarching findings documented in prior research and at the bivariate level in our own data were not apparent at the multivariate level. Our bivariate analyses explored overall relations between study variables whereas multivariate analyses aimed to examine unique variance in discrimination and mental health outcomes associated with overlapping identities. That is, by including predictors representing single demographic characteristics (e.g., Latinx) alongside terms representing overlapping identities (e.g., Latinx*gender), we endeavored to ascertain which variables significantly explained *unique* variance in our outcomes and, statistically, reduced the putative predictive power of main effects by including (and controlling for) interaction terms. Thus, the absence of significant main effects in multivariate models should not be interpreted as evidence that single demographic characteristics were unrelated to outcome variables as, in fact, they were at the bivariate level. Bivariate and multivariate results are therefore discussed together, below.

### Effects of demographics on experiences of discrimination

At the bivariate level, Latinx respondents indicated significantly higher discrimination in the workplace and characterized by exclusion and stigma; women reported higher discrimination characterized by exclusion and occurring in the workplace; and People of Color experienced greater discrimination, across PEDQ subscales, replicating prior research [24].

Our multivariate analyses expand upon this replication and evidence more complex relations evident when multiple demographics are taken into account at the same time. Indeed, main effects of Latinx ethnicity and gender on discrimination were noted and a significant interaction highlighted the unique experiences of Latinas in Texas. Regarding discrimination characterized by exclusion and occurring in the workplace, being a Latinx woman was associated with particular vulnerability—likely because items on these scales reflect the paternalistic power systems that have long been reported by Women of Color [21,60], including being ignored and othered. These experiences may be particularly pronounced for Latinx women who dress, speak, or behave in ways that differ from U.S. norms. Endorsement of items related to workplace and exclusionary discrimination may reflect the Latinx cultural value of *marianismo*—a female gender role associated with maintaining family interdependence and bonds, respect for societal hierarchies, and amicability in relationships— which can contribute to discrimination [61] Our findings add to a growing literature recognizing that Latinx women are subject to unique, stressors such as stereotyping related to “young mothers, hypersexual, being dependent on welfare, being domestic workers, and unintelligent” [61,64]. This finding builds on extant research on the double marginalization experienced by Women of Color; that is, their advancement in the workplace is thwarted by more than the “glass” ceiling of being a woman—they face the “concrete” ceiling of being a Woman of Color [62].

Regarding discrimination characterized by stigmatization, Latinx men exhibited the highest endorsement, echoing prior research [25,26,33] and indicating different experiences of discrimination in Latinxs depending on gender. Items measuring stigmatization in this study included “have others hinted that you are dishonest?”, “have others hinted that you must not be clean?”, “have people not trusted you?”, and “has it been hinted that you must be lazy?” These items mirror the xenophobic rhetoric that dominated media attention in the 2016 presidential race and is rearing its head once more in the current presidential race—that Latinx men are “drug dealers, criminals, rapists, etc.” [63]—and points to potential identity related stressors that may contribute to over representation in low-wage, strenuous, physical forms of employment, reinforce stereotypes of Latinos as lazy, dishonest, and physical laborers [64].

That neither main nor interactive effects of race or citizenship status on discrimination emerged should not be taken as evidence that these variables do not relate to discrimination. Indeed, at the bivariate level, both People of Color and non-citizens endorsed higher rates of discrimination, echoing what has been reported previously [29,36]. The absence of significant multivariate effects related to these variables likely reflect parceling of variance among related predictors (e.g., race and race*gender) as well as limitations in our sample size that forced combining meaningfully different categories relating to both race and citizenship.

### Effects of demographics and discrimination on mental health

Regarding mental health, women and non-citizens reported significantly higher symptoms than men and citizens, respectively. Neither Latinxs nor People of Color evidenced elevated mental health symptoms at the bivariate level, in contrast to prior research and hypotheses. Still, there is empirical support for the notion that college students of color and Latinxs under-report mental health symptoms, even when related concerns, like suicidal ideation, are reported [65]. Under-reporting could reflect cultural norms and stigma [65] or reflect mental health advantages as suggested by the Hispanic Health [66] and Immigrant [67] Paradoxes. Indeed, the relation between race/ethnicity and mental health outcomes is complex and the effect of risk factors, including discrimination, has been shown to be moderated by whether a young adult resides in an ethnic/racial enclave [68]. Our collapsing of People of Color into a single racial group ignored important within-group heterogeneity [45] and makes it difficult to fully unpack this finding, thus future research is warranted.

Multivariate analyses indicated a significant interaction between gender, race, and stigma-related discrimination on mental health, which suggests vulnerability for Women of Color. This interaction highlights the importance of considering intersectionality. In the multivariate model, main effects of gender and race were likely obscured by differences between men and Women of Color, but the significant interaction underscores the importance of race and gender in relation to mental health, nonetheless. Moreover, the interaction cautions against concluding that race is unrelated to mental health, as bivariates suggested. Instead, our findings mirror prior research indicating that relations between discrimination and psychological distress are stronger among Black women than Black men [35] and echo our findings related to Latinx women. Like Latinx women, Women of Color experience systemic barriers associated with their race, their gender, and their intersection. Not only do Women of Color have to contend with patriarchal power dynamics, limiting stereotypes about women, and stereotypes of People of Color, they also face additional, unique barriers including stereotypes associated being Women of Color specifically [62].

### Citizenship

At the bivariate level, non-citizen status was associated with significantly higher endorsement of discrimination as well as significantly higher mental health symptoms. These findings mirror documented vulnerability for undocumented young adults in terms of discrimination and mental health disparities [69]. The absence of citizenship effects in our multivariate models likely reflects limitations in our sample. Indeed, in this study, and in Texas more generally, most non-citizens are Latinx [70] and our sample had a relatively small number of non-Latinx, non-citizens (*n* = 69) for comparison, who were split along other demographic lines (e.g., race). In multivariate models we essentially queried the experiences of non-citizens that were not also associated with being Latinx—explanatory power in this regard would have been increased by a larger and more diverse sample. Moreover, a larger sample would also allow a nuanced analysis of citizenship, as small cell sizes forced collapsing meaningfully different groups. Unfortunately, many people are hesitant to report on their immigration status, particularly as anti-immigrant rhetoric and immigration enforcement actions are on the rise, likely accounting for missing data on that variable. Still, our bivariate findings should not be ignored, as they highlight vulnerability among non-citizen college students.

### Implications

Our results suggest that college students who identify as People of Color, Latinxs, women, and non-citizens experience the highest rates of discrimination within our sample. On the other hand, Women and non-citizens endorsed the highest mental health symptoms. However, experiences of discrimination associated with stigmatization were generally associated with deleterious mental health outcomes. This finding highlights a need for colleges and universities to utilize strategies targeting discrimination experienced by students through maintenance of Diversity, Equity, and Inclusion offices, cultural centers, supporting minoritized student organizations, mandating campus wide anti-discrimination trainings, and building a welcoming environment for marginalized students [42]. Another key finding from this research was that the greatest vulnerability for elevated mental health problems being among Latinx women experiencing stigma-related discrimination. This finding highlights limitations with single-axis analyses of experiences of discrimination [7] and emphasizes the importance of university wide mental health screening and interventions that account for layered forms of oppression. Further research is needed to identity mechanism contributing to Latinx women’s elevated risk for mental health problem in response to discrimination.

### Limitations & future directions

Several important limitations must be noted. First, we did not capture important identity dimensions: gender non-conforming individuals (*n* = 2) had to be excluded despite risk for discrimination and distress [71], and sexual orientation and socioeconomic status (SES) were not measured. Financial aid is a crude proxy for SES that is confounded with other aspects of identity—like citizenship status [72]. Existing research suggests that the factors we examined, like gender and race, influence the relation between discrimination and mental health differently depending on SES [37], warranting future research. It is important to note that anti-immigrant rhetoric and policies have continued to escalate in the U.S. since data were collected for the present study, which may impact the generalizability of our findings to Latinx college students navigating the current climate.

Despite the large sample, and efforts to keep cell sizes large by combining categories, additional interaction effects may have been found with more participants [73]. Further, our methodology may have obscured effects that would have emerged if we had used more nuanced methodologies, like asking respondents to rank the relative importance of identities and asking them to rank the “recognizability” of their identities to the outside world—important directions for future research. Indeed, several limitations hinge on the fact that we conceptualized demographic identities as individual categories—falling prey to the definitional issues of race/ethnicity outlined in previous research [74,75]. Additionally, our study made use of self-report variables and interrelations may have therefore represent shared method variance. Replication with multiple informants, diary designs, interviews, and greater breadth of mental health outcomes is needed.

Finally, it should be noted that our measure of discrimination relied on a designed to assess ethnic discrimination and modified to probe discrimination associated with membership in “your group,” broadly. Therefore, we are not able to disentangle whether discrimination was related to ethnicity or other demographics. Also, the PEDQ does not ask participants to specify the setting in which discrimination occurred, who perpetrated it, or how severe it was; examining these variables is essential in future research particularly in elucidating the extent to which anti-discrimination efforts should focus on campuses versus other public spheres. Finally, the Threat subscale suffered from non-normality and results should be interpreted with caution.

### Conclusions

We aimed to add to the existing literature on college students in Texas by probing demographic predictors of discrimination and mental health.Our findings suggest Latinx women are experiencing higher levels of discrimination than their counterparts and that experiences of discrimination are associated with increased mental health symptoms among Women of Color. These results indicate that discrimination should be seriously considered in college counseling, echoing extant research [38]. Additionally, findings highlight the continued importance of discrimination along “classic” lines like gender and race/ethnicity on a new generation. Despite the rapid growth of women in college [1], discrimination continues to affect college-aged women and warrant clinical attention and advocacy, particularly for Women of Color—Latinx women and non-Latinx women alike. Finally, the significant interactive effects in our models, even controlling for individual main effects, indicate that we must do more than simply control for one or more demographic variables because their interaction is uniquely predictive of discrimination and mental health. Indeed, our findings indicate that race and Latinx ethnicity acted with gender to compound risk for discrimination and its effects on mental health, pointing to the importance of considering identity in research and clinical practice.
